# Update in polycystic ovary syndrome: new criteria of diagnosis and treatment in Japan

**DOI:** 10.1007/s12522-013-0145-1

**Published:** 2013-04-16

**Authors:** Toshiro Kubota

**Affiliations:** ^1^ Comprehensive Reproductive Medicine, Graduate School Tokyo Medical and Dental University 1‐5‐45, Bunkyo‐ku 113‐8519 Tokyo Japan

**Keywords:** Diagnosis, Japan, New criteria, Polycystic ovary syndrome, Treatment

## Abstract

Polycystic ovary syndrome (PCOS) is the most frequent endocrine disorder in women of reproductive age. In 2006 the Japanese Society of Obstetrics and Gynecology (JSOG) proposed new, revised diagnostic criteria that in the future could also be valued internationally. Based on the new diagnostic criteria, the JSOG has also proposed the revised treatment criteria in 2008. In PCOS obese patients desiring children, weight loss and exercise is recommended. Nonobese patients, or those obese women who do not ovulate after lifestyle changes, are submitted to ovulation‐induction therapy with clomiphene citrate (CC). Obese CC‐resistant patients who have impaired glucose tolerance or insulin resistance are treated with a combination of metformin and CC. If these treatments options are unsuccessful, ovulation induction with exogenous gonadotropin therapy or laparoscopic ovarian drilling (LOD) is recommended. A low‐dose step‐up regimen is recommended with careful monitoring in order to reduce the risk of ovarian hyperstimulation syndrome (OHSS) and multiple pregnancies. Alternatively, with LOD high successful pregnancy rates of around 60 % are expected with a low risk of multiple pregnancies. If ovulation induction is unsuccessful, IVF‐ET treatment is indicated. In high OHSS‐risk patients, systematic embryo freezing and subsequent frozen embryo transfer cycles are recommended. In nonobese, anovulatory PCOS patients not desiring children, pharmacological treatments such as Holmström, Kaufmann regimens or low‐dose oral anticonceptives are used to induce regular withdrawal bleeding. These treatments are especially important for preventing endometrial hyperplasia and endometrial cancer. These new diagnostic and treatment criteria hopefully will contribute to an improved care of PCOS patients in Japan.

## Introduction

Polycystic ovary syndrome (PCOS) is a common endocrine disorder occurring in 5–10 % of women in reproductive age [1]. Hyperandrogenism, chronic anovulation, and infertility are the main features of this heterogeneous condition [2]. It affects female reproductive performance as well as it has effects on female health. It was originally described in 1935 by Stein IF and Levental ML as a syndrome consisting of oligomenorrhea and obesity with enlarged polycystic ovaries [3]. The diagnosis of PCOS is based on a combination of clinical, biological, and ultrasound criteria that have been used variably to define PCOS [4, 5]. Diagnosis criteria and PCOS definitions used by clinicians and researchers are almost as heterogenous as the syndrome. Currently, PCOS is defined by 2003 Rotterdam criteria [5], which requires at least two of three features for diagnosis: chronic anovulation, clinical and/or biochemical signs of hyperandrogenism, or polycystic ovaries. In Japan, compared to other countries, the usual clinical presentation of PCOS is slightly different, with less frequently encountered cases of hyperandrogenism, therefore established European or US guidelines are clinically less useful. In 2006, the Japanese Society of Obstetrics and Gynecology (JSOG) has proposed new, revised diagnostic criteria that in the future could also be valued internationally [6].

## Clinical data of PCOS in Japan

**Figure 1:**
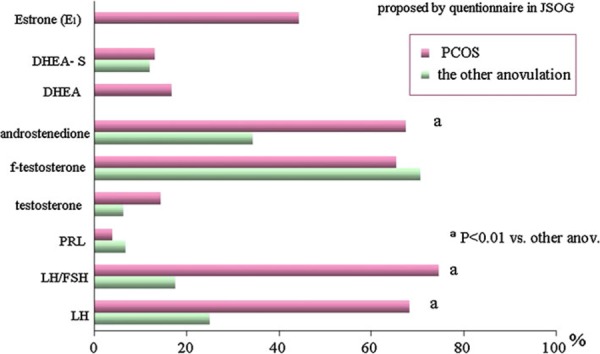
Rate of abnormal range of serum hormone levels in PCOS and the other ovarian dysfunction in Japan. Rates of abnormal range of androstenedione, LH/FSH and LH in PCOS are significantly higher than those in the other anovulation [6]

**Figure 2:**
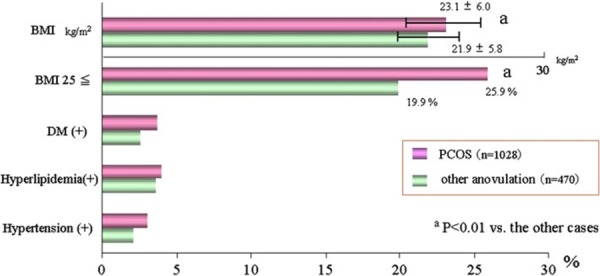
Obesity, DM and hyperlipidemia in PCOS; BMI of PCOS is significantly higher than that in the other. Rate of BMI higher than 25 kg/m^2^ is 25.9 % and significantly higher than that of the others [6]

**Figure 3:**
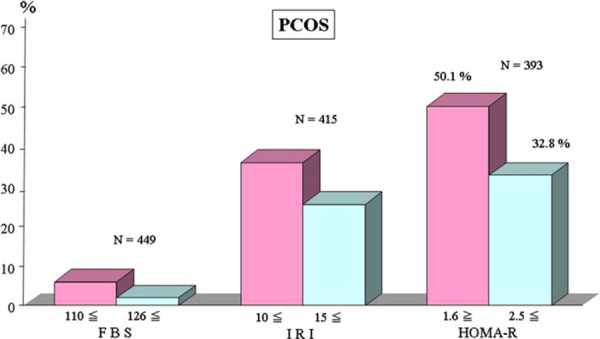
HOMA‐R, fasting blood sugar (FBS) and immunoreactive insulin (IRI) of PCOS in Japan; Rate of HOMA‐R 2.5 or higher values is 32.8 %. Rate of high insulin resistance is about 1/3 of PCOS in Japan [6]. (HOMA‐R = FBS × IRI ÷ 405)

**Figure 4:**
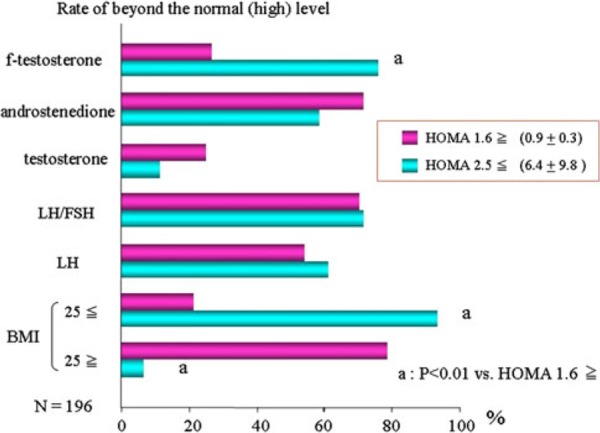
Serum hormone levels and insulin resistance in PCOS; PCOS with high HOMA‐R shows significantly higher values of free testosterone and BMI over 25 kg/m^2^ in Japan [6]

In 2005, JSOG performed the questionnaire research concerning about the clinical data of PCOS which were examined by 125 institutes or clinics in Japan. 1498 cases of ovarian dysfunction were divided into the PCOS group and the non‐PCOS group. Rates of abnormal range of serum hormone levels in PCOS and the other ovarian dysfunction in Japan are shown in Fig. 1 [6]. Rates of abnormal range of androstenedione, LH/FSH and LH in PCOS were significantly higher than those in the other anovulation (*P* < 0.01). Body mass index (BMI) values and frequency rates of obesity, DM and hyperlipidemia in the PCOS group are shown in Fig. 2. BMI values were 23.1 ± 6.0 kg/m^2^ in PCOS and 21.9 ± 5.8 kg/m^2^ in non‐PCOS, therefore the former was significantly higher than the latter (*P* < 0.01). Rate of BMI higher than 25 kg/m^2^ is 25.9 % in PCOS and significantly higher than that of the other group (*P* < 0.01). Figure 3 shows the rate of insulin resistance of PCOS in Japan. Rate of HOMA‐R higher than 2.5 is 32.8 %, and that of HOMA‐R lower than 1.6 is 50.1 %. So, rate of high insulin resistance is about 1/3 of PCOS in Japan. Serum hormone levels and insulin resistance in PCOS are shown in Fig. 4. PCOS with high HOMA‐R (≧2.5) shows significantly higher values (*P* < 0.01) of free testosterone and BMI over 25 kg/m^2^ in Japan [6].

## Clinical feature of PCOS

**Figure 5:**
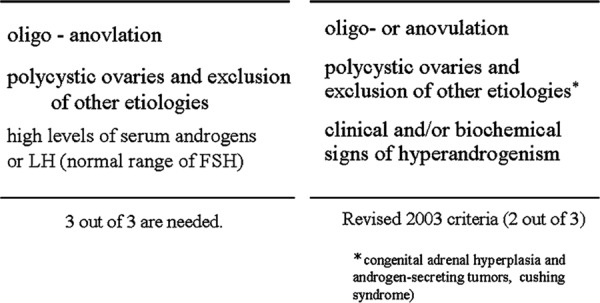
New criteria of PCOS in Japan and Europe/USA [5, 6]

Diagnostic criteria of PCOS is based on three main features: (1) cycle irregularities, (2) polycystic changes in the ovary by ultrasonography, (3) endocrine anomalies (LH or androgen hypersecretion). The diagnosis of PCOS was recently debated and suggestions followed in the Rotterdam Consensus statement [5] and in JSOG statement [6]. Figure 5 shows the diagnostic criteria of PCOS in Western countries and Japan.

Amenorrhea and oligomenorrhea are always present. Menstrual disturbances are present of obese PCOS and 72 % with lean PCOS. It is estimated that 40 to 60 % of women with PCOS in Western countries [7, 8] and 26 % of them in Japan [6] are overweight or obese. Obesity has been associated with infertility and an increased risk of menstrual irregularity or amenorrhea [9]. Transvaginal sonography (TVS) plays an essential role in diagnosis and treatment of PCOS. JSOG mentioned criteria for PCO, i.e., atypical polycystic pattern was defined by the presence of 10 or more cysts measuring 2–9 mm in diameter in a single plane arranged peripherally around stroma [6]. TVS has got important role in diagnosing, monitoring and treatment of PCOS patients. So careful clinical, biochemical, and TVS monitoring with tender loving care is required [10].

## Endocrine anomalies and insulin resistance in PCOS

### Serum hormone levels

Abnormal LH/FSH ratio is the main issue in the continuation of anovulatory state in PCOS subjects. Increased LH and decreased or normal FSH are due to (a) GnRH pulsatile secretion, i.e. at hypothalamic level. (b) high estrogen environment, i.e., at pituitary level [10]. In PCOS, clinically intense androgenization due to excess androgen production is observed. Hyperandrogenemia induces the increase in testosterone, androstenedione, dehydroepiandrosterone (DHEA), DHEA‐S, 17‐hydroxyprogesterone and estrone (E1) (excess androgen converted to E1 by peripheral fat). Decrease in the sex hormone binding protein in the liver, increase in insulin response in the ovary and the effect of high LH, induce the increase in androgen secretion in the ovary. After that, follicle growth and maturation are suppressed [5, 6, 11].

### Insulin resistance and concomitant hyperinsulinemia

Insulin resistance and concomitant hyperinsulinemia are frequently found in obese PCOS women [11, 12]. Approximately 50–60 % of PCOS patients suffer from insulin resistance in Western countries [10] and 33 % in Japan [6]. Seventy % of obese PCOS and 20 % of thin PCOS have hyperinsulinemia in Western countries. Increased insulin resistance causes hyperglycemia leading to hyperinsulinemia and it amplifies LH action on theca cells and again increase in androgen level [10, 13]. Hyperinsulinemia, insulin resistance, and an increase in androgen production are all linked together in PCOS patients. It is also known that patients with insulin resistance are often resistant to ovulation induction [10, 12].

## New criteria of PCOS treatment in Japan

**Figure 6:**
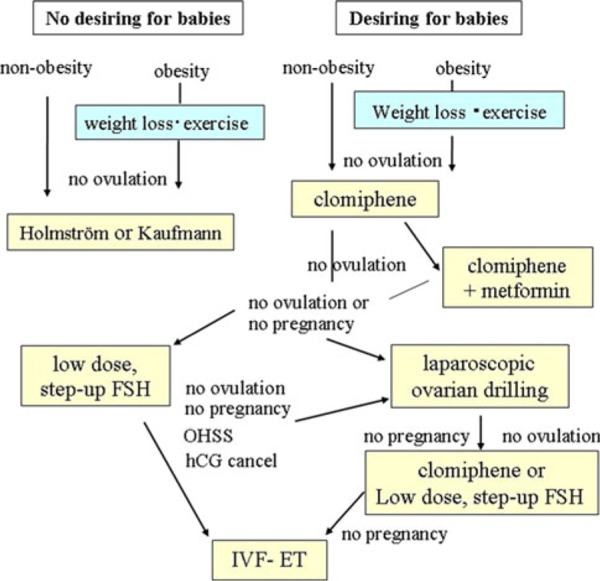
New criteria of PCOS treatment in Japan [14]

Based on the above‐mentioned diagnostic criteria, the JSOG has also proposed the revised treatment criteria of PCOS in 2008 (Fig. 6) [14].

### Treatment of PCOS desiring children

#### Treatment of obesity in PCOS

For obese PCOS patients (BMI ≧25 kg/m^2^), weight loss and exercise are recommended as a first option. Norman et al. [15]. demonstrated that lifestyle modification led to increased insulin sensitivity and resulted in improved ovulation and fertility in obese women with PCOS. This approach of lifestyle modification, which includes weight‐reducing diet and exercise, should be the first step in the management of them [16]. Increase in physical activity and loss of at least 10 % of body weight are given in the form of lifestyle modification. Weight reduction causes spontaneous ovulation and dose of stimulation of medicine is less required [17, 18]. Lifestyle management should also be used as the primary therapy in overweight and obese women with PCOS for the treatment of metabolic complications [19].

#### Clomiphene citrate for PCOS

Clomiphene citrate (CC) is the standard drug used for ovulation induction in women with PCOS [20, 21, 22, 23, 24]. Successful ovulation is achieved in 70–85 % of them and 40–50 % will conceive [25]. Nonobese patients or those obese women who do not ovulate after lifestyle changes are submitted to ovulation induction therapy with CC. CC is antiestrogenic and it binds with estrogen receptors causing decreased concentration of receptors. So activation of GnRH, increase in FSH and/or LH and growth of the dominant follicle occur. All cycles of CC should be monitored by ultrasound. To prevent ovarian hyperstimulation syndrome (OHSS), we should have control over the growing follicle and E2 levels. Hyperandrogenism and obesity affect the CC response adversely. Failure to ovulate with 3 months of use of CC 150 mg/day for 5 days is called CC resistance, and 20 % of PCOS patients will be CC resistant [10]. In CC resistant PCOS patients with hirsutism and high androgen concentrations, the combination of dexamethasone and CC is effective, because dexamethasone reduces the levels of androgens [26]. In CC resistant PCOS patients with galactorrhea or hyperprolactinemia, both CC and dopamine agonists should be used [27].

#### Combination of metformin and clomiphene citrate(CC) for PCOS with insulin resistant

Numerous articles have been published where insulin sensitizers such as biguanides (metformin) [28, 29, 30] and thiazolidinediones (troglitazone) [31] have been used and proven to improve metabolic abnormalities in PCOS patients. Metformin declines the peripheral glucose level without interfering with pancreatic β‐cell function [10], by inhibiting hepatic gluconeogenesis and increasing insulin receptor affinity. Metformin reduces levels of LH, hyperinsulinemia and decrease ovarian levels of androgen [32]. Hyperinsulinemic and hyperandrogenic PCOS patients with thecal hyperplasia are best downregulated with metformin. Several trials were prospective double‐blind placebo controlled [33, 34, 35, 36]. Each of those trials randomized metformin with placebo in the CC‐resistant patient. In one trial [35] there was no difference in outcome. The other trials had a statistically significant improvement when metformin was added to CC in the CC‐resistant patients. Kurabayashi et al. [37] also reported that combination of metformin and CC could improved ovulation rates in CC‐resistant infertile Japanese women with PCOS in spite of no effect of metformin treatment alone. Therefore, obese CC‐resistant PCOS patients who have impaired glucose tolerance or insulin resistance are treated with a combination of metformin and CC [38].

#### Ovulation induction of gonadotropin therapy in PCOS

Ovulation induction with exogenous gonadotropin therapy is recommended for unsuccessful patients in the previously mentioned treatments. During ovulation induction, a chronic, low‐dose step‐up regimen of FSH‐only preparations is recommended [39, 40] with careful monitoring in order to reduce the risk of OHSS [41] and multiple pregnancies. Purified FSH has a theoretical advantage of avoiding additional LH in PCOS. It is always better to start with the smallest dose, and always be watchful on vaginal ultrasonography for number of follicles stimulated and how they are growing. But E2 level should always be kept suitable prior to hCG injection when there is evidence of multiple follicles [10]. If 4 or more numbers of follicles are found to be getting ≧16 mm diameter, hCG injection should be canceled [14, 42]. These results demonstrate that the low‐dose step‐up regimen for with PCOS is the safest protocol among the stimulation regimens for reducing multiple follicular development.

#### Laparoscopic ovarian drilling (LOD) in PCOS

The history of management of PCOS has been sharp turns from surgical management to medical therapy and later a renewed interest in surgery [43]. Gjonnaess proposed that ovulation was initiated by either stromal destruction or extensive capsular destruction with discharge of the contents of multiple follicle cysts [44]. At present, LOD is indicated in clomiphene resistant cases and another approach of gonadotropin therapy [45]. LOD is an effective procedure in properly selected cases, because drilling appears to be equally effective with lesser chances of multiple pregnancies [45, 46]. In LOD treatment, high success pregnancy rates of around 60 % are expected after treatment within 6 months of time with a low risk of adverse effects in PCOS, and peak pregnancy rate is seen around 6–9 months after surgery [47].

The exact mechanism of induction of ovulation by LOD is not understood. This may be attributed to the improved intraovarian stromal blood flow following the procedure [43]. Daniell and Miller [48] suggested that physical opening of subcapsular cysts led to the removal of androgen‐containing follicular fluid from the ovarian environment, thus lowing the androgen content of ovaries. The total and free testosterone is decreased to 40–50 % of the preoperative levels. LH levels also decreased following the procedure. Change in FSH levels is less marked and normal inhibin pulsality is restored. The normalization of hormonal relationships leads to recruitment of a new cohort of follicles and resumption of ovarian function. These endocrine changes occur rapidly and are sustained for years [43]. The number of holes to be drilled depends upon the size of the ovaries and the sonographic appearance which had been noted during the preoperative work‐up. In moderately enlarged ovaries, about 10–12 holes are sufficient but more may be required in voluminous ovaries [43]. Treatment patients when followed sonographically show spontaneous ovulation or much more improved sensitivity to CC [45]. Overall LOD is simple procedure with lots of benefits for fertility preservation, but it should be judiciously employed with strict selection protocol.

#### IVF‐ET treatment in PCOS

If ovulation induction is unsuccessful or conception cannot be accomplished as mentioned above, IVF‐ET treatment is indicated. PCOS has been associated with various negative effects on ovulation induction and IVF‐ET outcomes [49, 50, 51, 52, 53, 54]. During ovarian stimulation for IVF‐ET, the low‐dose step‐up regimens of pure FSH preparation are used for women with PCOS to prevent adverse effects [39, 40]. In order to achieve ovulation in those patients, it needs ovarian stimulation with more risk for OHSS [41]. In high OHSS‐risk patients, systematic embryo freezing and subsequent frozen embryo transfer cycles are recommended [49, 55].

In vitro maturation (IVM) of oocyte procedure would be a best option for PCOS treatment in terms of complete prevention of OHSS [56]. Since the technology has been clinically applied, the number of centers undergoing it has increasing. In IVM procedure, the immature oocytes in germinal vesicle or metaphase I stage are cultured with medium, gonadotropins and serum for 24–48 h. About 50–60 % of immature oocytes commonly mature to metaphase II stage [57]. In order to acquire better outcome, it is essential to elucidate maturation process of human oocytes.

### Treatment of PCOS not desiring for children

For obese PCOS patients, weight loss and exercise are recommended [17, 18, 19]. In non‐obese and anovulatory patients, pharmacological treatments such as Holmström, Kaufmann regimens or low‐dose oral anticonceptives are used to induce regular withdrawal bleeding. Early detection of PCOS mitigate the risks of endometrial hyperplasia and cancer [9], which caused by ‘unopposed estrogen’ condition of PCOS. There are moderate quality data to support that women with PCOS have a 2.7‐fold increased risk for endometrial cancer [58]. Most endometrial cancers are known to be well differentiated and have a good prognosis. These hormone therapies are especially important for preventing endometrial hyperplasia and endometrial cancer [59].

## Conclusion

New, revised diagnostic criteria and revised treatment criteria of PCOS reported by JSOG are proposed in this review. These new criteria hopefully will contribute to an improved care of PCOS patients in Japan.

## Acknowledgment

We have no conflict of interest.

### Open Access

This article is distributed under the terms of the http://creativecommons.org/licenses/by/3.0/ License which permits any use, distribution, and reproduction in any medium, provided the original author(s) and the source are credited.

## References

[1] Azziz R , Woods KS , Reyna R , Key TJ , Knochenhauer ES , Yildiz BO . The prevalence and feature of the polycystic ovary syndrome in an unselected population. J Clin Endocrinol Metab, 2004, 89, 2745–2749 10.1210/jc.2003-032046 15181052

[2] Franks S . Polycystic ovary syndrome. N Engl J Med, 1995, 333, 853–861 10.1056/NEJM199509283331307 7651477

[3] Stein IF , Leventhal ML . Amenorrhoea associated with bilateral polycystic ovaries. Am J Obstet Gynecol, 1935, 29, 181–191

[4] Zawadzki JK , Dunaif A Dunaif A Givens JR , Haseltine FP , Merriam GR . Diagnostic criteria for polycystic ovary syndrome: toward a rational approach. Polycystic ovary syndrome, 1992 Boston Blackwell Scientific 377–384

[5] The Rotterdam ESHRE/ASRM‐Sponsored PCOS Consensus Workshop Group . Revised 2003 consensus on diagnostic criteria and long‐term health risks related to polycystic ovary syndrome (PCOS). Hum Reprod, 2004, 19, 41–47 10.1093/humrep/deh098 14688154

[6] The Japanese Society of Obstetrics and Gynecology (JSOG). Constitute of Reproductive rndocrinology. Reports of a new diagnostic criteria of PCOS in Japan. Acta Obstet Gynecol Jpn. 2007;59:868–86.

[7] Carmina E , Legro RS , Stamets K , Lowell J , Lobo RA . Difference in body weight between American and Italian women with polycystic ovary syndrome: influence of the diet. Hum Reprod, 2003, 18, 2289–2293 10.1093/humrep/deg440 14585875

[8] Ogden CL , Yanovski SZ , Carroll MD , Flegal KM . The epidemiology of obesity. Gastroenterol, 2007, 132, 2087–2102 10.1053/j.gastro.2007.03.052 17498505

[9] Santoro N . Update in hyper‐ and hypogonadotropic amenorrhea. J Clin Endocrinol Metab, 2011, 96, 3281–3288 10.1210/jc.2011-1419 22058375

[10] Deshmukh S. Polycystic ovarian syndrome. In: Allahbadia GN, editors. Infertility management made easy. Anshan Ltd: Tunbridge Wells; 2007. p. 172–97.

[11] Dunaif A . Insulin resistance and the polycystic ovarian syndrome; mechanism and implications for pathogenesis. Endocrinol Rev, 1997, 18, 774–800 10.1210/er.18.6.774 9408743

[12] Chang RJ , Nakamura RM , Judd HL , Kaplan SA . Insulin resistance in non obese patients with polycystic ovarian disease. J Clin Endocrinol Metab, 1983, 57, 356–359 10.1210/jcem-57-2-356 6223044

[13] Homburg R . Androgen circle of polycystic ovary syndrome. Hum Reprod, 2009, 24, 1548–1555 10.1093/humrep/dep049 19279033

[14] The Japanese Society of Obstetrics and Gynecology (JSOG). Constitute of reproductive endocrinology. Reports of a new treatment criteria of PCOS in Japan. Acta Obstet Gynecol Jpn. 2009;61:902–12.

[15] Norman RJ , Davies MJ , Lord J , Moran IJ . The role of lifestyle modification in polycystic ovary syndrome. Trends Endocrinol Metab, 2002, 13, 251–257 10.1016/S1043-2760(02)00612-4 12128286

[16] Clark AM , Thornley B , Tomlinson L , Galletley C , Norman RJ . Weight loss in obese infertile women results in improvement in reproductive outcome for all forms of fertility treatment. Hum Reprod, 1998, 13, 1502–1505 10.1093/humrep/13.6.1502 9688382

[17] Kiddy DS, Hamilton‐Fairley D, Bush A, Short F, Anyaoku V, Reed MJ. Improvement in endocrine and ovarian function during dietary treatment of obese women with polycystic ovary syndrome. Clin Endocrinol (Oxf). 1992;36:105–11. 10.1111/j.1365-2265.1992.tb02909.x 1559293

[18] Teixerira PJ , Going SB , Houtkooper LB , Cussler EC , Metcalfe LL , Blew RM . Pretreatment predictors of attrition and successful weight management in women. Int J Obes Relat Metab Disord, 2004, 28, 1124–1133 10.1038/sj.ijo.0802727 15263921

[19] Moran LJ , Pasquali RP , Teede HJ , Hoeger KM , Norman RJ . Treatment of obesity in polycystic ovary syndrome: a position statement of the androgen excess and polycystic ovary syndrome society. Fertil Steril, 2009, 92, 1966–1982 10.1016/j.fertnstert.2008.09.018 19062007

[20] Shepard MK , Balmaceda JP , Leija CG . Relationship of weight to successful induction of ovulation with clomiphene citrate. Fertil Steril, 1979, 32, 641–645 510566 10.1016/s0015-0282(16)44411-0

[21] O'Herlihy C , Pepperell RJ , Brown JB , Smith MA , Sandri L , McBain JC . Incremental clomiphene therapy: a new method of treating persistent anovulation. Obstet Gynaecol, 1981, 58, 535–542 6795551

[22] Lobo RA, Gysler M, march CM, Goebelamann U, Mishell DR Jr. Clinical and laboratory predictors or clomiphene response. Fertil Steril. 1982;37:168–74. 7199484

[23] Legro RS , Barnhart HX , Schlaff WD , Carr BR , Diamond MP , Carson SA et al. Clomiphene, metformin, or both for infertility in the polycystic ovary syndrome. N Engl J Med, 2007, 356, 551–566 10.1056/NEJMoa063971 17287476

[24] Neveu N , Granger L , St‐Michel P , Lavoie HB . Comparison of clomiphene citrate, metformin, or the combination of both for first‐line ovulation induction and achievement of pregnancy in 154 women with polycystic ovary syndrome. Fertil Steril, 2007, 87, 113–120 10.1016/j.fertnstert.2006.05.069 17081535

[25] The ESHRE Capri workshop . Female infertility: treatment options for complicated cases. Hum Reprod, 1997, 12, 1191–1196 10.1093/humrep/12.6.1191 9222000

[26] Brown J, Farquhar C, Beck J, Boothroyd C, Hughes E. Clomiphene and anti‐oestrogens for ovulation induction in PCOS. Cochrane Database Syst Rev. 2009(4):CD002249. 10.1002/14651858.CD002249.pub4 19821295

[27] Bracero N , Zacur HA . Polycystic ovary syndrome and hyperprolactinemia. Obst Gynecol Clin North Am., 2001, 28, 77–84 10.1016/S0889-8545(05)70186-8 11293005

[28] Velazquez EM , Mendoza SG , Hamer T , Soga F , Glueck CJ . Metformin therapy in polycystic ovary syndrome reduces hyperinsulinemia, insulin resistance, hyperandrogenaemia and systolic blood pressure, while facilitating normal menses and pregnancy. Metabolism, 1994, 43, 647–654 10.1016/0026-0495(94)90209-7 8177055

[29] Nestler JE, Stovall D, Alchter N, iuorno MJ, Jakubowicz MJ. Strategies for the use of insulin‐sensitizing drugs to treat infertility in women with polycystic ovary syndrome. Fertil Steril. 2002;77:209–15. 10.1016/s0015-0282(01)02963-6 11821072

[30] Seli E , Duleba AJ . Optimizing ovulation induction in women with polycystic syndrome. Curr Opin Obstet Gynaecol, 2000, 14, 245–254 10.1097/00001703-200206000-00002 12032379

[31] Erhmann D , Schneider DJ , Sobel BE , Cavaghan MK , Imperial J , Sturis J . Troglitazone improves defects in insulin action, insulin secretion, ovarian steroid genesis, and fibrinolysis in women with polycystic ovary syndrome. J Clin Endocrinol Metab, 1997, 82, 2108–2116 10.1210/jc.82.7.2108 9215280

[32] Nestler JE , Jakubowics D . Lean women with polycystic ovary syndrome respond to insulin reduction with decreases in ovarian P450c17 alpha activity and serum androgens. J Clin Endocrinol Metab, 1997, 82, 4075–4079 10.1210/jc.82.12.4075 9398716

[33] Kocak M, caliskan E, Simsir C, Haberal A. Metformin therapy improves ovulatory rates, cervical scores, and pregnancy rates in clomiphene citrate‐resistant women with polycystic ovary syndrome. Fertil steril. 2002;77:101–6. 10.1016/s0015-0282(01)02941-7 11779598

[34] Vandermolen DT , Ratts V , Evans WS , Stovall DW , Kauma SW , Nester JE . Metformin increases the ovulatory rate and pregnancy rate from clomiphene citrate in patient with polycystic ovary syndrome who is resistant to clomiphene citrate alone. Fertil Steril, 2001, 75, 310–315 10.1016/S0015-0282(00)01675-7 11172832

[35] Ng EH, Wat NM, Ho PC. Effects of metformin on ovulation rate, hormonal and metabolic profiles in women with clomiphene‐resistant polycystic ovaries: a randomized, double‐blind placebo‐controlled trial. Hum Reprod. 2001;16:1625–31. 10.1093/humrep/16.8.1625 11473953

[36] Sturrock NDC , Lannon B , Fay TN . Metformin does not enhance ovulation induction in clomiphene resistant polycystic ovary syndrome in clinical practice. Br J Pharmacol, 2002, 53, 469–473 10.1046/j.1365-2125.2002.01575.x PMC1874363 11994052

[37] Kurabayashi T , Suzuki M , Kashima K , Banzai J , Terabayashi K , Fujita K , Tanaka K . Effects of low‐dose metformin in Japanese women with clomiphene‐resistant polycystic ovary syndrome. Reprod Med Biol, 2004, 3, 19–26 10.1111/j.1447-0578.2004.00047.x PMC7187776 32351315

[38] Siebert TI , Kruger TF , Steyn DW , Nosarka S . Is the addition of metformin efficacious in the treatment of clomiphene citrate‐resistant patients with polycystic ovary syndrome? A structured literature review. Fertil Steril, 2006, 86, 1432–1437 10.1016/j.fertnstert.2006.06.014 17007847

[39] Nugent D, Vandekerckhove P, Hughes E, Arnot M, Lilford R. Gonadotrophin therapy for ovulation induction in subfertility associated with polycystic ovary syndrome. Cochrane Database Syst Rev. 2000(4):CD000410. 10.1002/14651858.CD000410 11034687

[40] Homburg R , Levy T , Ben‐Rafael Z . A comparative prospective study of conventional regimen with chronic low‐dose administration of follicle‐stimulation hormone for anovulation associated with polycystic ovary syndrome. Fertil Steril, 1995, 63, 729–733 7890055 10.1016/s0015-0282(16)57473-1

[41] Nabot D , Bergh PA , Laufer N . Ovarian hyperstimulation syndrome in novel reproductive technologies: prevention and treatment. Fertil Steril, 1992, 58, 249–261 1633889 10.1016/s0015-0282(16)55188-7

[42] Mathur RS , Akande AV , Keay SD , Hunt LP , Jenkins JM . Distinction between early and late ovarian hyperstimulation syndrome. Fertil Steril, 2000, 73, 901–907 10.1016/S0015-0282(00)00492-1 10785214

[43] Jain N. Laparoscopic surgery in infertility and gynecology. In: Gomel V editor. Ovarian drilling. Tunbridge Wells: Anshan Ltd; 2011. p 142–55.

[44] Gjonnaess H . Polycystic ovarian syndrome treated by ovarian electrocautery through the laparoscope. Fertil Steril, 1984, 41, 20–25 6692959 10.1016/s0015-0282(16)47534-5

[45] Farquhar CM , Williamson K , Gudex G , Johnson NP , Garland J , Sadler L . A randomized controlled trial of laparoscopic ovarian diathermy versus gonadotropin therapy for women with clomiphene citrate‐resistant polycystic ovary syndrome. Fertil Steril, 2002, 78, 404–411 10.1016/S0015-0282(02)03225-9 12137881

[46] Farquhar C, Liford RJ, Marjoribanks J, Vandekerckhove P. Laparoscopic ‘drilling’ by diathermy or laser for ovulation induction in anovulatory polycystic ovary syndrome. Cochrane databases Syst Rev. 2007(3):CD001122. 10.1002/14651858.CD001122.pub3 17636653

[47] Amer SA , Banu Z , Li TC , Cooke ID . Long‐term follow‐up of patients with polycystic ovary syndrome after laparoscopic ovarian drilling: endocrine and ultrasonographic outcomes. Hum Reprod, 2002, 17, 2815–2817 10.1093/humrep/17.11.2851 12407038

[48] Daniell JF , Miller W . Polycystic ovaries treated by laparoscopic laser vaporization. Fertil Steril, 1989, 51, 232–236 2912769 10.1016/s0015-0282(16)60482-x

[49] D'Angelo A, Amso N. Embryo freezing for preventing ovarian hyperstimulation syndrome. Cochrane Database Syst Rev. 2007;CD002806. 10.1002/14651858.CD002806.pub2 PMC11513185 17636707

[50] Homburg R . Polycystic ovary syndrome: induction of ovulation. Baillieres Clin Endocrinol Metab, 1996, 10, 281–292 10.1016/S0950-351X(96)80127-3 8773749

[51] Mulders AG , Laven JS , Imai B , Eijkemans MJ , Fauser BC . IVF outcome in anovulatory infertility (WHO group 2)—including polycystic ovary syndrome—following previous unsuccessful ovulation induction. Reprod Biomed Online, 2003, 7, 50–58 10.1016/S1472-6483(10)61728-2 12930574

[52] Hamilton‐Fairley D , Kiddy D , Watson H , Paterson C , Frank S . Association of moderate obesity with a poor pregnancy outcome in women with polycystic ovary syndrome treated with low dose gonadotropin. Br J Obstet Gynaecol, 1992, 99, 128–131 10.1111/j.1471-0528.1992.tb14470.x 1554664

[53] Fedorcsak P , Dale PO , Storeng R , Tanbo T , Abyholm T . The impact of obesity and insulin resistance on the outcome of IVF or ICSI in women with polycystic ovarian syndrome. Hum Reprod, 2001, 16, 1086–1091 10.1093/humrep/16.6.1086 11387273

[54] Wang JX , Davis MJ , Norman RJ . Polycystic ovarian syndrome and the risk of spontaneous abortion following assisted reproductive technology treatment. Hum Reprod, 2001, 16, 2606–2609 10.1093/humrep/16.12.2606 11726582

[55] D'Angelo A, Amso N: “Coasting” (withholding gonadotrophins) for preventing ovarian hyperstimulation syndrome. Cochrane Database Syst Rev. 2002;CD002811. 10.1002/14651858.CD002811 12137659

[56] Trounson A , Wood C , Kausche A . In vitro maturation and the fertilization and developmental competence of oocytes recovered from unstimulated polycystic patients. Fertil Steril, 1994, 62, 353–362 8034085 10.1016/s0015-0282(16)56891-5

[57] Morimoto Y. Cytoplasmic maturation and mitochondrial activity in human IVM. Abstract of the 16th world Congress on In Vitro Fertilization. In: Concurrent symposium C‐14. 2011; p. 170, September Tokyo, Japan.

[58] O'Connor KA , Ferrell R , Brindle E , Trumble B , Shofer J , Holman DJ , Weinstein M . Total and unopposed estrogen exposure across stages of the transition to menopause. Cancer Epidemiol Biomark Prev, 2009, 18, 828–836 10.1158/1055-9965.EPI-08-0996 PMC2675575 19240232

[59] Korytkowski MT , Mokan M , Horwitz MJ , Berga SL . Metabolic effects of oral contraceptives in women with polycystic ovary syndrome. J Clin Endocrinol Metab, 1995, 80, 3327–3334 10.1210/jc.80.11.3327 7593446

